# Spontaneous Intramural Oesophageal Haematoma in a Patient with Uncontrolled Hypertension: An Unusual Chest Pain Aetiology

**DOI:** 10.1155/2017/4086056

**Published:** 2017-02-20

**Authors:** Samantha Cooray, Dionysios Dellaportas, Clifford Caruana, Andrew R. Davies

**Affiliations:** Department of Upper Gastrointestinal Surgery, St. Thomas' Hospital, Guy's and St. Thomas' Oesophago-Gastric Centre, London, UK

## Abstract

*Introduction*. Spontaneous intramural oesophageal haematoma is a rare condition that usually occurs secondary to an acute or chronic coagulation disorder. The presenting complaint is often with retrosternal chest pain and most patients are initially investigated to exclude more common causes in the differential diagnosis, such as acute coronary syndromes. Severe life-threatening bleeding or perforation seldom, if ever, arises.* Case Presentation*. We present a case of spontaneous oesophageal haematoma which appears to have developed gradually in a 69-year-old female with uncontrolled hypertension and antiplatelet medication use. The diagnosis was made on computed tomography imaging and was further evaluated with upper gastrointestinal endoscopy. Management was conservative and a follow-up endoscopy two weeks later showed almost complete resolution of the lesion.* Discussion*. Spontaneous oesophageal haematomas are very rare and usually result in the separation of the mucosal layer from the underlying muscle, presenting with chest pain, haematemesis, and dysphagia. Usually the diagnosis is one of exclusion, based on computed tomography imaging and endoscopy. Conservative management is almost always successful.

## 1. Introduction

Oesophageal haematoma is a rare condition that normally occurs as a result of severe vomiting or trauma, either accidental or iatrogenic [1]. More infrequently it can be spontaneous and in such cases is usually associated with coagulopathy disorders or the use of anticoagulation agents [2]. Oesophageal haematomas are almost twice as prevalent in middle age women compared to men, although the reasons for this are unclear. Management is usually conservative and most hematomas resolve spontaneously. Complications such as severe bleeding or perforation are rare [3].

## 2. Case Presentation

A 69-year-old lady presented to the emergency department with sudden onset, severe crushing central chest pain radiating to the back, followed by a single episode of vomiting. She had no haematemesis, dysphagia, or odynophagia and no history of trauma. Her past medical history included a known small hiatus hernia, hypercholesterolaemia, and migraines. She was taking clopidogrel for small vessel cerebrovascular disease and had also recently been started on amlodipine for hypertension.

On initial assessment, the patient was haemodynamically stable with a heart rate (HR) of 62 beats per min, a respiratory rate (RR) of 20/min, oxygen saturation of 99% on room air, and a temperature of 36.3°C and was hypertensive with blood pressure (BP) 204/110 mmHg. On clinical examination she had some chest wall tenderness with bruising seen over the suprasternal notch and upper part of the sternum. Cardiovascular, respiratory, and abdominal examinations, however, were unremarkable. Haemoglobin was 11.5 g/dL. All other routine laboratory tests were normal including troponin < 20, INR 1.0, and APTT ratio 0.9. An electrocardiogram (ECG) and chest X-ray were also unremarkable.

**Figure 1:**
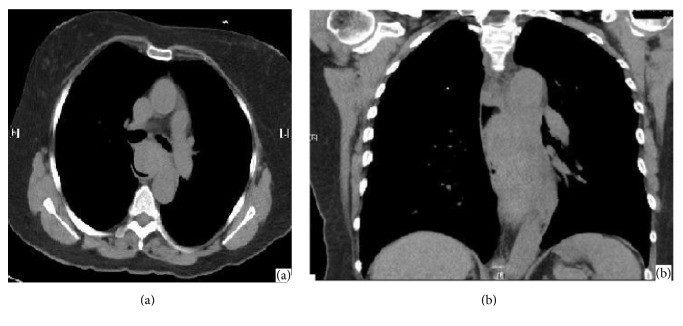
Initial CT demonstrating the long intramural oesophageal haematoma ((a) sagital and (b) coronal view).

A computed tomography (CT) aortogram was urgently performed to exclude the presence of an aortic dissection or aneurysm. This revealed a distal intramural oesophageal haematoma 36 mm in diameter with proximal oesophageal dilatation, extending down to the gastroesophageal junction (Figure 1). On the basis of the CT scan, a diagnosis of a spontaneous oesophageal haematoma was made, with the only potentially contributing factors being hypertension and the use of clopidogrel.

Oesophageal perforation was excluded from the absence of clinical or biochemical parameters for sepsis, pneumomediastinum, pleural effusion, or contrast extravasation on cross-sectional imaging. The patient was managed conservatively in a High Dependency Unit (HDU) for the first 48 hours. Clopidogrel was stopped; oral diet restricted and intravenous (IV) fluids, analgesia, antiemetics, and proton-pump inhibitors (PPI) were administered regularly.

**Figure 2:**
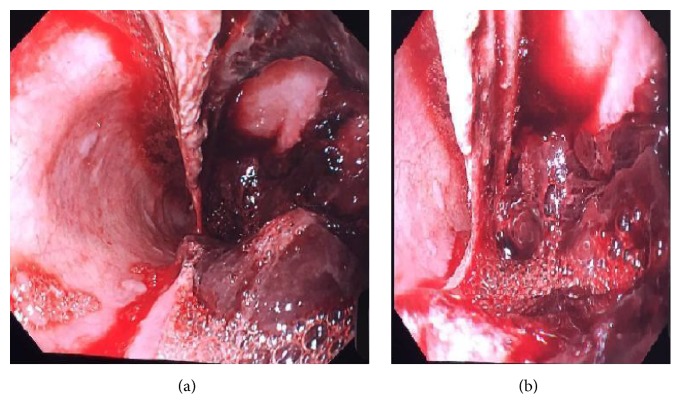
Endoscopic view of intramural oesophageal haematoma projecting into the lumen.

An upper gastrointestinal endoscopy demonstrated an anterolateral intramural haematoma at 20 cm extending to the gastrooesophageal junction (Figure 2), with no obvious underlying oesophageal mucosal lesion. Random oesophageal biopsies showed acute and chronic inflammation of the squamous mucosa extending into the subepithelial tissue with fibrinous debris containing* Candida* spores and hyphae. At the time of the endoscopy, a nasojejunal (NJ) feeding tube was placed endoscopically to facilitate enteral nutrition bypassing the haematoma.

**Figure 3:**
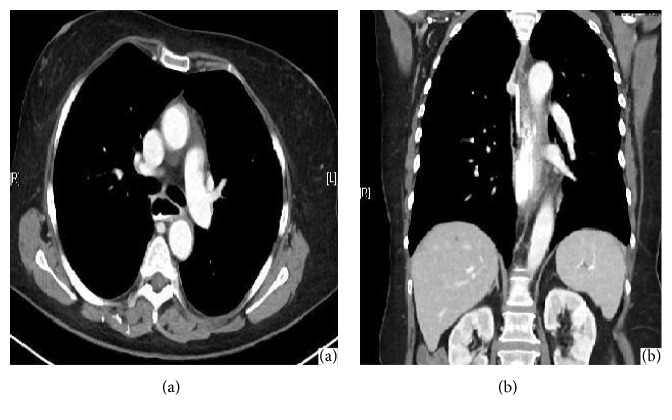
Follow-up CT showing resolving haematoma ((a) sagital and (b) coronal view).

The hypertension was controlled with regular amlodipine, and the oesophageal* Candida* infection was treated with IV fluconazole. On careful history questioning, it was revealed that the patient's blood pressure was poorly controlled, and careful monitoring was applied, and normal blood pressure was achieved. Although oesophageal candidiasis was treated, it cannot be pointed out as the aetiology of the condition. A repeat CT of the chest with IV and oral contrast ten days later demonstrated the haematoma had largely resolved (Figure 3). Oral intake was increased as tolerated.

**Figure 4:**
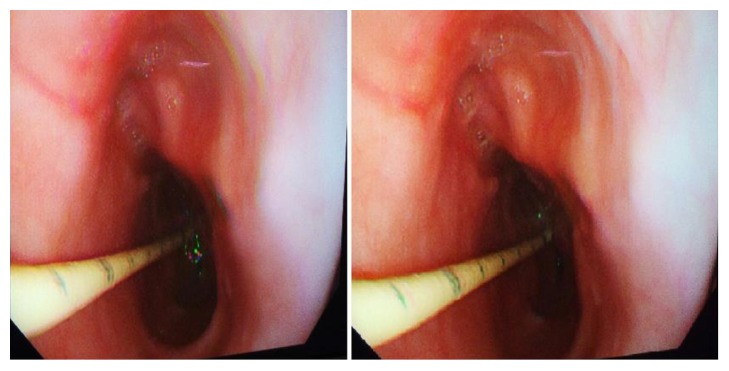
Follow-up endoscopic view of resolved haematoma-nasojejunal tube shown in situ.

The patient was discharged with the NJ tube in situ and maintained on a regular dose of PPI. On repeat upper gastrointestinal endoscopy two weeks later, the haematoma had completely resolved and the oesophageal mucosa appeared normal, excluding the presence of an underlying malignancy or any other predisposing lesion (Figure 4). On follow-up three months later, the patient remains symptom-free.

## 3. Discussion

Spontaneous oesophageal haematomas (SOH) are very rare and usually result in the separation of the mucosal layer from the underlying muscle [4]. The predominant symptom is chest pain, which may be accompanied by haematemesis and dysphagia, forming a triad of characteristic symptoms [5]. However, as with case presented here, patients may present with only one of these symptoms [6]. This not only makes diagnosis difficult but also highlights the importance of differentiating it from other serious conditions such as acute coronary syndrome and aortic dissection.

Usually the diagnosis of SOH is one of exclusion and is based on CT imaging and endoscopy, after other more routine tests have failed to identify a cause [7]. The most common finding on CT is referred to as the “double barrel” sign where there is the appearance of a double lumen within the oesophagus. An elongated soft tissue-like mural mass, narrowing the oesophageal lumen, may also be seen [8]. Endoscopy usually identifies friable, discoloured mucosa in the mid and distal oesophagus, but the reason for this location is unclear. It has been speculated that the oesophagus has less support from adjacent structures in this anatomical area [5].

The management of SOH is usually conservative. Patients are kept nil by mouth initially and maintained on IV fluids and NG/NJ feeding, with gradual reintroduction of a soft diet. In the majority case studies reported to date, patient outcomes have been good with complete resolution of lesions and no long-term complications [9]. In extremely rare cases, angiography and embolization or surgical intervention is mentioned, probably when a distinctive oesophageal pathology is eventually identified [10].

The aetiology of SOH is not well understood, but it is thought to be clinically distinct from other oesophageal disorders such as Mallory-Weiss tears and Boerhaave's syndrome [11]. Mallory-Weiss tears usually present with haematemesis as the predominant feature and patients with Boerhaave's syndrome tend to be haemodynamically unstable and septic, which is not the case with SOH. In addition, SOH occurs in older age groups and is more common in women than the other conditions [12]. It has been suggested by some that Mallory-Weiss tears may predispose to SOH. However, SOH can occur in the absence of vomiting or retching that typically precedes such tears. In SOH there is usually an association with coagulopathies or the use of antiplatelet or anticoagulant agents [13]. This was evident in the case presented here where the patient was taking long-term clopidogrel. She also had uncontrolled hypertension, and although not often cited as a contributing factor, a number of case reports mention hypertension as part of the past medical history [11, 12]. Considering this patient complained of chest pain three weeks prior to presentation, the haematoma may have been of gradual onset possibly from a small capillary leak, exacerbated by the uncontrolled hypertension and antiplatelet therapy. As reported for spontaneous haematomas located in other parts of the gastrointestinal tract, coagulopathies and anticoagulation treatment are the main underlying factors, and management is mostly conservative [14]. For oesophageal haematomas though, any surgical intervention would be of extremely high risk and should be avoided.

In summary, SOH needs to be considered in the differential diagnosis of acute chest pain. Management in specialised oesophagogastric units is strongly advised. Despite the unclear aetiological mechanisms, prognosis is excellent with conservative treatment.
